# Construct validity of the Physiotherapy Evidence Database (PEDro) quality scale for randomized trials: Item response theory and factor analyses

**DOI:** 10.1002/jrsm.1385

**Published:** 2020-01-05

**Authors:** Emiliano Albanese, Lukas Bütikofer, Susan Armijo‐Olivo, Christine Ha, Matthias Egger

**Affiliations:** ^1^ Faculty of BioMedicine Università della Svizzera italiana Lugano Switzerland; ^2^ Faculty of Medicine University of Geneva Geneva Switzerland; ^3^ Institute for Social and Preventive Medicine (ISPM) University of Bern Bern Switzerland; ^4^ CTU Bern University of Bern Bern Switzerland; ^5^ Faculty of Rehabilitation Medicine, Department of Physical Therapy University of Alberta Edmonton AB Canada; ^6^ Faculty of Business and Social Sciences Universityof Applied Sciences Osnabrück Germany

**Keywords:** item response theory, physiotherapy, randomized clinical trials, risk of bias, study quality scale, validation

## Abstract

**Background:**

There is an agreement that the methodological quality of randomized trials should be assessed in systematic reviews, but there is a debate on how this should be done. We conducted a construct validation study of the Physiotherapy Evidence Database (PEDro) scale, which is widely used to assess the quality of trials in physical therapy and rehabilitation.

**Methods:**

We analyzed 345 trials that were included in Cochrane reviews and for which a PEDro summary score was available. We used one‐ and two‐parameter logistic item response theory (IRT) models to study the psychometric properties of the PEDro scale and assessed the items' difficulty and discrimination parameters. We ran goodness of fit post estimations and examined the IRT unidimensionality assumption with a multidimensional IRT (MIRT) model.

**Results:**

Out of a maximum of 10, the mean PEDro summary score was 5.46 (SD = 1.51). The allocation concealment and intention‐to‐treat scale items contributed most of the information on the underlying construct (with discriminations of 1.79 and 2.05, respectively) at similar difficulties (0.63 and 0.65, respectively). The other items provided little additional information and did not distinguish trials of different quality. There was substantial evidence of departure from the unidimensionality assumption, suggesting that the PEDro items relate to more than one latent trait.

**Conclusions:**

Our findings question the construct validity of the PEDro scale to assess the methodological quality of clinical trials. PEDro summary scores should not be used; rather, the physiotherapy community should consider working with the individual items of the scale.

**Highlights**

What is already known

- There is a debate and variation in practice between different fields over how to assess quality and the potential for bias in randomized trials.

What is new

- This is the first comprehensive and independent construct validation study of a widely used quality scale, the Physiotherapy Evidence Database (PEDro) scale using item response theory models in a large sample of physiotherapy trials. Our findings show that the PEDro scale to assess the quality of randomized trials has poor construct validity, with items capturing more than one underlying trait.

Potential impact

- The research synthesis community should agree on a common theoretical framework and approach to assessing quality and risk of bias in trials that are both valid and consistent. The use of summary scores to screen and select randomized trials in physical therapy and other fields should be discouraged. Rather, reviewers should assess different domains of bias separately, as recommended by the Cochrane Collaboration.

## 1 INTRODUCTION

Systematic reviews and meta‐analyses of randomized trials have a pivotal role informing clinical practice and policy decisions,^1^ and there is a broad agreement that the methodological quality of primary studies should be carefully assessed. However, study quality is a hazy concept that lacks a commonly agreed definition and a solid theoretical framework: Many of the available tools to assess study quality lack both theoretical and empirical support.^2^, ^3^, ^4^ The quality of randomized trials was originally defined as “the confidence that the [study] design, conduct, and analysis has minimized or avoided bias.”^5^ In line with this definition, the Cochrane Collaboration distinguishes between the methodological quality of a study and the risk of bias: A study of high quality can still be at high risk of bias.^6^ For example, in physiotherapy and other nonpharmaceutical interventions, the blinding of study participants may be impossible even in studies that otherwise meet high methodological standards. The Cochrane risk of bias (RoB) tool exclusively focuses on the internal validity of trials.^6^, ^7^ Others extend quality assessment to include elements of external validity and the precision of estimates or sample size or to items related to the completeness of the reporting of trials.^8^

The impact of study quality or risk of bias on the results of trials has been studied for different scales and checklists (ie, criterion and/or convergent validity)^9^, ^10^, ^11^ but evidence to support construct validity is sparse. Although the empirical demonstration of construct validity is strictly not possible, evidence is needed to establish not only the salience of existing measures to the study quality construct but also the extent to which study quality is a coherent concept. Moreover, there is little recognition that, from a test theory viewpoint, there are important differences between scales and checklists.^12^ Their interchangeable use is problematic, for example, when checklists are turned into scales simply by assigning 1 point to every item, and overall scores are computed.^13^ Scales use several items to assess one underlying construct (a latent trait) that cannot be directly observed, for example, “study quality.” The combination of individual responses into an overall score is meaningful only if all items relate to the same latent construct (unidimensionality) and are correlated indicators of this construct (internal consistency), rather than variables causing the construct.^12^, ^14^

In contrast to scales, checklists may relate to different constructs, and they may include both indicators of effects of the underlying construct and indicators of causes of the construct.^14^ For example, the Cochrane RoB tool assesses the blinding or lack of blinding of study participants and personnel, which may prevent or cause performance bias.^6^, ^7^ Similarly, it assesses concealment of allocation to treatment, which may prevent selection bias. Of note, while the latter can always be implemented in a trial, the former may be impossible; consequently, the correlation between the two is often low. These items should therefore not be combined in a scale and summary score. Another problem of summary scores relates to the implicit assumption that all scale items contribute equally to the overall score, whereas in practice, their importance and correlation with the underlying construct vary and will depend on the type of intervention and outcome, and context in general.^2^, ^15^ Cutoffs along the continuum of summary scores are often used to denote “adequate quality” and to decide on inclusion or exclusion of studies in systematic reviews and meta‐analyses, which may introduce bias.^16^, ^17^

In this study, we focus on the Physiotherapy Evidence Database (PEDro) scale, which is widely used to assess the methodological quality of clinical trials in the field of physical therapy and rehabilitation. The development and evaluation of the PEDro scale have been exceptionally meticulous.^18^, ^19^ However, unsurprisingly, there is debate about the pitfalls of using summary scores to assess study quality and risk of bias.^17^, ^20^ Although modern validation studies increasingly use item response theory (IRT) to examine the discrimination of different items included in a scale and their coverage of the latent (underlying) construct,^21^, ^22^ no such studies have been performed for the PEDro scale. We therefore examined the construct validity of the PEDro scale using IRT models in a large sample of physiotherapy trials.^16^

## 2 METHODS

### 2.1 The PEDro scale

**Figure 1:**
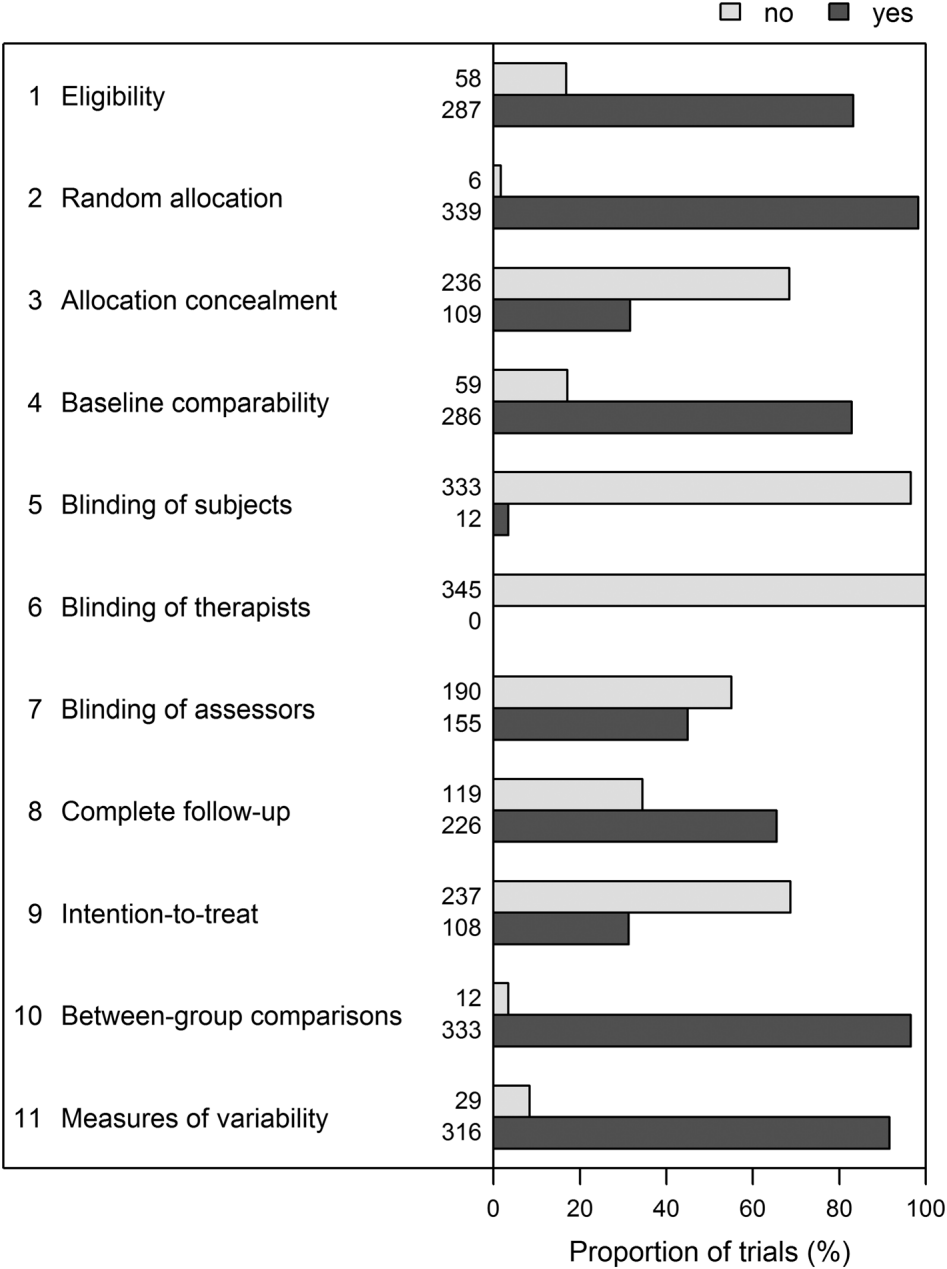
Items of the Physiotherapy Evidence Database (PEDro) scale and their distribution in the validation dataset of 345 trials. Item 1 (eligibility criteria) does not contribute to the total score

PEDro^23^ is a web‐based repository of currently over 42 000 RCTs of physical therapy that have been systematically assessed by two independent reviewers using the PEDro scale.^24^ Figure 1 details the 11 items included in the PEDro scale. Eight items relate to the design and conduct of the trial, and three are concerned with reporting eligibility criteria (item 1), between‐group statistical comparisons (item 10), and measures of variability (item 11). Notably, only two of the three items on reporting quality contribute points to the total score: The item on eligibility criteria does not. Therefore, the summary score ranges between 0 and 10 rather than 11 points, and usually trials are regarded to be of moderate or high quality if they score six points or more.^24^ The PEDro scale was developed for clinical trials of physical therapy. However, it does not contain items that are specific to this field,^17^, ^18^ and it has been used in other fields, for example, in reviews of drug interventions in dementia or pain.^23^, ^25^

**Table 1:** Trial characteristics by Physiotherapy Evidence Database (PEDro) scores of below 6 or 6 and above

	All Trials	PEDro Score <6	PEDro Score ≥6	*P* Value*
Number of trials	345	188	157	
PEDro score	5.00 (4.00‐7.00)	5.00 (4.00‐5.00)	7.00 (6.00‐7.00)	<.001
Year of publication	2002 (1998‐2004)	2000 (1997‐2004)	2003 (1999‐2005)	<.001
Trial design				.25
Parallel	322 (93%)	174 (93%)	148 (94%)	
Factorial	15 (4.3%)	7 (3.7%)	8 (5.1%)	
Crossover	7 (2.0%)	6 (3.2%)	1 (0.6%)	
Cluster	1 (0.29%)	1 (0.53%)	0 (0.0%)	
Multicentric study	85 (25%)	30 (16%)	55 (35%)	<.001
Hypothesis				.41
Superiority	317 (92%)	169 (90%)	148 (94%)	
Equivalence	15 (4.3%)	11 (5.9%)	4 (2.5%)	
Noninferiority	4 (1.2%)	2 (1.1%)	2 (1.3%)	
Unclear/no comparison	9 (2.6%)	6 (3.2%)	3 (1.9%)	
Placebo‐controlled	28 (8.1%)	9 (4.8%)	19 (12%)	.017
Study population				.20
Adult	218 (63%)	122 (65%)	96 (61%)	
Geriatric	116 (34%)	57 (30%)	59 (38%)	
Pediatric	5 (1.4%)	4 (2.1%)	1 (0.6%)	
Unclear	6 (1.7%)	5 (2.7%)	1 (0.6%)	
Interventions				.031
Exercise	266 (77%)	154 (82%)	112 (71%)	
Education	9 (2.6%)	5 (2.7%)	4 (2.5%)	
Manual therapy	13 (3.8%)	4 (2.1%)	9 (5.7%)	
Acupuncture	8 (2.3%)	1 (0.53%)	7 (4.5%)	
Other	49 (14%)	24 (13%)	25 (16%)	
Outcome source				.036
Clinician assessment	128 (37%)	82 (44%)	46 (29%)	
Self‐reported	180 (52%)	86 (46%)	94 (60%)	
Laboratory data	15 (4.3%)	9 (4.8%)	6 (3.8%)	
Administrative data	22 (6.4%)	11 (5.9%)	11 (7.0%)	
Sample size				
Randomized	72.0 (41.0‐148)	59.0 (32.0‐109)	104 (60.0‐191)	<.001
Analyzed^a^	63.0 (37.0‐128)	49.0 (30.0‐95.0)	93.0 (54.0‐170)	<.001
Funding^b^				
Industry	36 (10%)	17 (9.0%)	19 (12%)	.86
Government	183 (53%)	79 (42%)	104 (66%)	.002
Academic	41 (12%)	23 (12%)	18 (11%)	.50
Foundation	96 (28%)	46 (24%)	50 (32%)	.70
No funding	18 (5.2%)	12 (6.4%)	6 (3.8%)	.22
Not declared	69 (20%)	50 (27%)	19 (12%)	.001

*Note.* Numbers (%) or medians (interquartile range) are shown.

a Unknown for 10 trials with a PEDro score <6 and 1 trial with a PEDro score ≥6.

b Does not add up to 100% as multiple entries are possible.

* *P* values obtained from Wilcoxon rank‐sum and Fisher exact tests for continuous and categorical variables, respectively.

### 2.2 Study sample

Described in detail elsewhere,^16^ we analyzed 345 physiotherapy trials that were included in systematic reviews published in the Cochrane Database of Systematic Reviews (CDSR). Briefly, we searched the CDSR from 1 January 2005 to 25 May 2011 for meta‐analyses of physical therapy interventions. Meta‐analyses were eligible if they included at least three trials of physiotherapy as defined by the World Confederation for Physical Therapy (WCPT) with a continuous outcome.^26^ A PEDro score was already available in the online PEDro database for 333 of the 345 trials (94.3%). Thus, almost all trials of our sample were independently assessed by two independent PEDro reviewers.^19^ The 12 remaining RCTs were assessed by two independent assessors, who were trained by an experienced meta‐analyst (S.A‐O), with 100% agreement between the two assessors who scored the 12 RCTs.^16^

### 2.3 Statistical methods

We used Wilcoxon rank‐sum tests and Fisher exact tests to compare the trials of moderate to high quality (PEDro score ≥6) with trials of lower quality for continuous and categorical variables, respectively. We assessed correlation of each item with the summary PEDro score (including all items) using Pearson correlation coefficients and the internal consistency based on Cronbach alpha and its standardized version, Guttman lambda 6, the averaged between‐item correlation (mean and median), and the signal‐to‐noise ratio. To address potential multidimensionality, we used a stratified version of Cronbach alpha and McDonald omega assuming one, two, or three underlying dimensions.

We computed a series of IRT models to study the psychometric properties of the PEDro scale. In IRT models, the relationship between the PEDro scale's dichotomous item responses (no = 0; yes = 1) and the underlying latent trait (RCT quality) are described by the item characteristic curve (ICC). For each scale item, the ICC displays the probability of responding “yes” in relation to the latent trait θ, the study quality. This probability follows a cumulative logistic distribution and increases as the latent trait increases, ie, the probability for a “yes” increases with study quality. The latent trait is on a standard normal scale, ie, 95% of the studies are expected to have a quality between −1.96 and 1.96. The ICC is characterized by two parameters, the item difficulty (or location) and the discrimination. The latter is assumed to be identical for all items in one‐parameter logistic (1PL) models (which corresponds de facto to the classical Rash model) but varies across items in two‐parameter logistic (2PL) models. The item difficulty reflects the study quality that is required to have a 50‐50 chance of responding “yes.” The discrimination is the slope of the ICC and captures how well an item can distinguish between different levels of the latent trait around the item difficulty. An item with a large discrimination (and a steep ICC) is answered differently for studies of different quality.

We ran standard 1PL, and 2PL IRT models for PEDro items, and a 2PL multidimensional IRT (MIRT) model with two‐dimensions (2D 2PL). We compared the goodness of fit of these models using likelihood ratio tests and global fit statistics including the Akaike information criteria (AIC), the Bayesian information criteria (BIC), the M2 statistic,^27^ the root mean square error of approximation (RMSEA), the standardized root mean square residual (SRMSR), the Tucker‐Lewis index (TLI), and the comparative fit index (CFI). We analyzed item fit based on a signed chi‐squared statistic and the RMSEA.^28^ For the unidimensional models, we also calculated infit and outfit mean square statistics that focus on the differences near or at the extreme of the θ values, respectively. We also assessed person‐fit using the person‐fit based on the Zh value^29^ (for more details, see Tables S8, S9, S10, and S11).

We calculated the ICC and the item information functions (IIF) of all items, where “information” refers to the precision of a scale in measuring the latent trait, and each item has greatest precision around its estimated difficulty parameter. Technically, the information is the negative of the expectation of the second derivative of the log‐likelihood with respect to the latent trait θ. We used the item information functions to depict the coverage and precision of the items with respect to the spectrum of the clinical trials' quality. We included item 1 of the PEDro scale (eligibility criteria) in all IRT models.

IRT models are based on two key assumptions: that the scale items draw on only one underlying latent trait (unidimensional latent space) and that the item responses are independent and conditional only to the level of the underlying trait (local or conditional independence). We formally tested the former assumption comparing the multidimensional 2D 2PL model with the unidimensional models (Appendix S1, section 1.3). We assessed local independence for all IRT models using the local dependence statistic between each pair of items (a signed chi‐squared value) and its standardized version (Cramer V) (Appendix S1, section 1.5).^30^

All analyses were done in Stata version 14 (StataCorp, College Station, Texas) and R (R Foundation for Statistical Computing, Vienna, Austria), using Stata routines *irt 1PL* and *irt 2PL*, and the R *MIRT* packages (2D 2PL model).

## 3 RESULTS

### 3.1 Characteristics of sample of 345 physiotherapy trials

As shown in Figure 1, the counts of yes/no responses varied markedly across the 11 items of the PEDro scale. For example, blinding of subjects (item 5) and of therapists (item 6) were virtually never implemented, while items related to random allocation of participants to groups (item 2), reporting of between‐group comparisons (item 10) or reporting of both point estimates and measures of variability (item 11) were almost always met. Consequently, only 10 trials (3%) had either a very low (<3) or very high score (>8), and most quality scores ranged between 4 and 7 with a maximum of 10 (median = 5, interquartile range = 4‐7, mean = 5.46, SD = 1.51). Of note, item 1 (eligibility criteria), which was not used in the calculations of the overall score, showed results similar to item 4 (baseline comparability). Trials with PEDro scores below six points differed from those with higher PEDro scores (Table 1). The latter were published more recently, and were more likely to be multicentric trials and placebo controlled. Trials with higher PEDro scores also had larger sample sizes and were more likely to report the source of funding and to be funded by government grants.

### 3.2 Internal consistency

Some of the items correlated only weakly with the summary PEDro score (Table S20), in particular, item 1 (eligibility, Pearson correlation coefficient (*ρ*) = .29; 95% confidence interval [CI], 0.19‐0.38), item 2 (random allocation, *ρ* = .2; 95% CI, 0.10‐0.30), item 5 (blinding of subjects, *ρ* = .29; 95% CI, 0.19‐0.38), and item 10 (between‐group comparisons, *ρ* = .19; 95% CI, 0.09‐0.29). Consequently, internal consistency of the PEDro items was low (Cronbach *alpha* = .56; 95% CI, 0.50‐0.63; stratified Cronbach *α* with two dimensions = .59; McDonald *omega* with two dimensions = .58) with a mean correlation of 0.11 (Table S21).

### 3.3 Model fit

The two‐parameter logistic (2PL) and the two‐dimensional two‐parameter logistic (2D 2PL) models showed a reasonable global fit (eg, M2 of 42.8, *P* = .17, and 18.6, *P* = .85; Table S6), whereas the one‐parameter (1PL) model did not (eg, M2 of 74.2, *P* = .003). The 2PL model fitted better than the 1PL model (*P* = .001 from likelihood ratio test), indicating that the assumption of a common discrimination does not hold. All models struggled to fit items 2 (random allocation) and 5 (blinding of subjects), which showed a very high and a very low proportion of positive responses, respectively.

### 3.4 Results from two‐parameter logistic model

**Figure 2:**
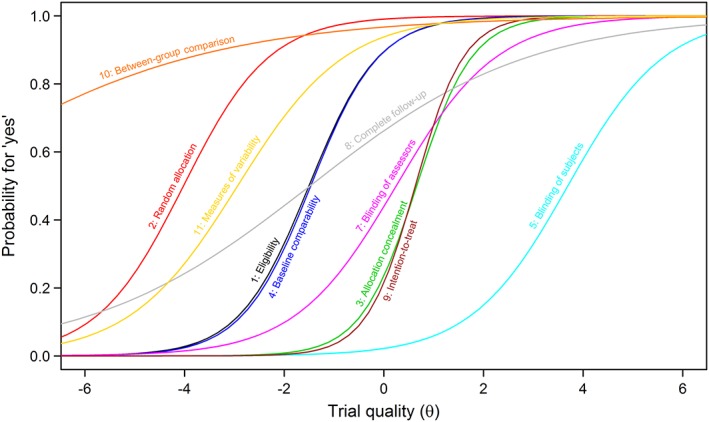
Item characteristic curves from the two‐parameter logistic model [Colour figure can be viewed at http://wileyonlinelibrary.com]

**Figure 3:**
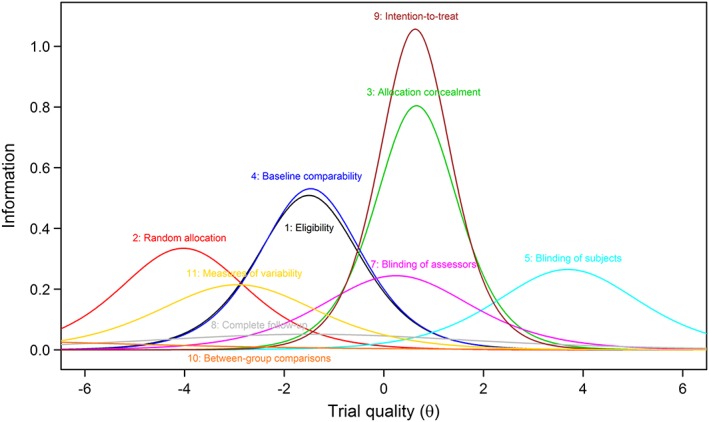
Item information functions (IIF) from the two‐parameter logistic model for all PEDro items. Items 3 and 9 contributed the most information but at the same trial quality. The coverage for qualities larger than 2 was poor [Colour figure can be viewed at http://wileyonlinelibrary.com]

Table 2 shows the difficulty and discrimination coefficients from the 2PL model, Figure 2 the item characteristics curves (ICC), and Figure 3 the item information functions (IIF). Results from the 1PL and the 2D 2PL models are presented in Tables S1 and S3, and Figures S1 and S2, respectively. In the 2PL model, the difficulty coefficient of items 2 (random allocation), 10 (between‐group comparisons), 11 (variability measures), and 5 (blinding of subjects) were either highly negative (below −2.9, ie, “too easy”) or highly positive (above 3.6, ie, “too hard”) and thus contributed little information on the quality of trials in the normal range. Interestingly, these items all loaded on the same latent trait in the 2D 2PL model (Table S5), indicating that they relate to another latent trait.

**Table 2:** Items of the Physiotherapy Evidence Database (PEDro) scale with the coefficients for difficulty and discrimination from the item response theory two‐parameter logistic model

Item No.	Description	Label	Difficulty (95% CI)	Discrimination (95% CI)
1^a^	Eligibility criteria were specified	Eligibility	−1.50 (−1.98 to −1.03)	1.43 (0.76 to 2.09)
2	Subjects were randomly allocated to groups	Random allocation	−4.02 (−7.25 to −0.80)	1.16 (−0.05 to 2.36)
3	Allocation was concealed	Allocation concealment	0.66 (0.43 to 0.89)	1.79 (1.08 to 2.51)
4	The groups were similar at baseline regarding the most important prognostic indicators	Baseline comparability	−1.47 (−1.93 to −1.01)	1.46 (0.78 to 2.13)
5	There was blinding of all subjects	Blinding of subjects	3.69 (1.34 to 6.04)	1.03 (0.20 to 1.86)
6	There was blinding of all therapists who administered the therapy	Blinding of therapists	Not estimatable^b^	Not estimatable^b^
7	There was blinding of all assessors who measured at least one key outcome	Blinding of assessors	0.25 (−0.02 to 0.51)	0.99 (0.59 to 1.39)
8	Measures of at least one key outcome were obtained from more than 85% of the subjects initially allocated to groups	Complete follow‐up	−1.48 (−2.53 to −0.43)	0.45 (0.15 to 0.76)
9	All subjects for whom outcome measures were available received the treatment or control condition as allocated or, where this was not the case, data for at least one key outcome was analyzed by “intention to treat”	Intention‐to‐treat	0.63 (0.42 to 0.85)	2.05 (1.15 to 2.96)
10	The results of between‐group statistical comparisons are reported for at least one key outcome	Between‐group comparisons	−9.37 (−28.16 to 9.42)	0.36 (−0.39 to 1.11)
11	The study provides both point measures and measures of variability for at least one key outcome	Measures of variability	−2.94 (−4.47 to −1.40)	0.93 (0.33 to 1.53)

a Item 1 (eligibility criteria) does not contribute to total score.

b The parameters were not estimatable for item 6 because blinding of therapist was never implemented.

The slopes of the ICCs for items 8 (complete follow‐up) and 10 (between group comparison) were both flat, indicating that these items cannot distinguish well between trials of different quality level. For item 8, that was also true for the 2D 2PL model, where the item failed to discriminate between the two latent traits. Most of the information was provided by item 3 (allocation concealment) and item 9 (intention‐to‐treat analysis) with the highest discriminations (1.79 and 2.05, respectively); however, these two items had almost identical difficulty (0.63 and 0.65, respectively) and thus conveyed similar information regarding the underlying construct (ie, quality of trials). These items mainly loaded on the second latent trait in the 2D 2PL model.

### 3.5 Dimensionality and local independence

The 2D 2PL fitted better than the unidimensional 2PL model, indicating that the PEDro scale may rely on more than one underlying latent trait or dimension (Tables S6 and S7; all fit indices were improved and *P* = .004 in a likelihood ratio test). In the 2D 2PL model, items 1 (eligibility criteria, which is not computed for the overall score), 3 (allocation concealment), and 9 (intention‐to‐treat) loaded on one dimension and items 2 (random allocation), 5 (blinding of subjects), 10 (between group comparison), and 11 (point and variability measure) on the other (Table S5). Items 4 (balanced at baseline) and 7 (blinding of assessors) showed cross‐loading on both dimensions, whereas item 8 (complete follow‐up) struggled to load on any of them. Finally, we found evidence for local dependence for six combinations of items in the 1PL model and for one only in the 2PL model (items 3 and 7) and for none in the 2D 2PL model (Tables S12 to S14).

## 4 DISCUSSION

We conducted an independent construct validation study based on IRT models to assess the psychometric properties of a scale that is widely used to measure the quality of clinical trials in physical therapy and rehabilitation and to determine inclusion or exclusion of trials in systematic reviews and meta‐analyses. We validated the instrument in a large “real‐world” sample of trials that were both included in Cochrane reviews and assessed in the PEDro database. We found that the scale items used to compute the PEDro study quality score captured more than one underlying trait. Some items seemed to convey similar, limited, or no information about the methodological quality of the clinical trials. Our results corroborate earlier criticisms of quality scales in general^2^, ^15^, ^31^ and of the PEDro scale in particular.^16^, ^17^

Strengths of the present study include the use of IRT models, which go beyond previously used Rasch models,^32^ and the independence of our group, which is not associated with the PEDro database and scale. Several limitations are worth noting. Because we used a sample of 345 highly relevant trials that were included in Cochrane reviews, their average study quality was higher than the average reported in the PEDro online archive. Although the main features of our sample did not differ from those of trials included in the PEDro repository, we acknowledge that our validation study might produce different results in different groups of trials. In other words, it is unclear whether the lack of construct validity extends to trials of low quality. Difficulty and discrimination estimates were outside the typical range for some of the items and showed large uncertainty, which was attributable to a low frequency of negative or positive responses. These point estimates therefore have to be interpreted with care. We did not include multidimensional IRT models with more than two dimensions as they might be susceptible to overfitting for an 11‐item scale. The likely number of underlying dimensions remains unclear. However, an in‐depth analysis of the structure of underlying latent traits was beyond the scope of this study. Based on our results from the 2D 2PL model, it seems likely that items 1, 3, and 9 and items 2, 5, 10, and 11 relate to distinct latent traits, with items 4 and 7 loading on both of those traits.

Evidence on the construct validity of the PEDro scale is scarce and comparisons with previous studies not straightforward. Previous studies used simple linear regressions^11^, ^33^ or Rasch analysis^32^ to assess construct validity. However, linear regressions will only provide information about criterion validity, not on construct validity. Although the Rasch model corresponds to a 1PL IRT model, we found that the 1PL did not fit our data well. Our main results are based on the 2PL model where difficulty and discrimination parameters are allowed to vary across items. Further, in contrast to de Morton et al,^32^ there were important departures from the unidimensionality assumption. Replications in other samples are warranted, but these departures are unlikely to depend on the RCTs included in our study.

We found evidence of violations of local independence (the second assumption of IRT models), which were likely due to redundancy of items. Although item reduction was done during the development phase of the PEDro scale, future studies should consider whether two or more items in the current version are linked and whether there is any “carryover” from one item to the next, both of which cause violations of the local independence assumption.

The PEDro database, which includes many thousands of carefully assessed trials, is an extremely valuable resource for the evaluation of interventions in physical therapy and rehabilitation.^34^ In many respects, the development of the PEDro quality scale was exceptionally thorough.^8^ However, the PEDro scale intentionally includes two sets of items that capture internal validity (ie, “believability,” items 2 to 9) and reporting quality (“interpretability,” items 10 and 11), respectively. While item 1 does not contribute to the score, items that do not relate to the same underlying construct by design do contribute. In addition, the operationalization of some of the items seems problematic. For example, item 4 is a composite item, which enquires about both similarity between groups and prognostic indicators, and judgement for item 8 is based on the (implicit) assumption that less than 15% overall attrition is unproblematic, which is questionable and unsubstantiated. Further studies support the reliability^18^ and convergent validity of the PEDro scale.^33^ Nevertheless, our results question the construct validity of the PEDro scale in its current form and, therefore, support recommendations of Cochrane and many methodologists that the use of summary scores should be discouraged.^2^, ^6^, ^15^, ^31^, ^35^

In conclusion, our study provides robust empirical evidence to suggest that the PEDro scale as currently constructed and used is not psychometrically sound and should not be used to assess study quality. The PEDro instrument might be improved by removing redundant items, by revising others, and by clarifying the different underlying concepts of risk of bias, study quality, and completeness of reporting. The PEDro database and physical therapy community should now consider working with the assessments of the individual items of the scale, revising some of these items taking into account recent developments,^7^, ^35^ and refrain from computing and using summary scores. Finally, our results are relevant to the evidence synthesis community beyond PEDro, because they clearly demonstrate that we should agree urgently on an approach to assessing quality and RoB in trials that is both valid and consistent.

## CONFLICT OF INTEREST

The authors reported no conflict of interest.

## Supporting information

Data S1. Supporting information S1

## Data Availability

We analyzed 345 physiotherapy trials that were included in systematic reviews published in the Cochrane Database of Systematic Reviews (CDSR).
