# Supplementary material for: Construct validity of the Physiotherapy Evidence Database (PEDro) quality scale for randomized trials: Item response theory and factor analyses
Source: Res Synth Methods. 2020 Jan 5;11(2):227–36. doi: 10.1002/jrsm.1385 (PMC7079093; doi:10.1002/jrsm.1385)
Supplement: Supplementary file 1 — Data S1. Supporting information S1 [file JRSM-11-227-s001.pdf]

# Appendix

## Construct validity of the Physiotherapy Evidence Database (PEDRo) quality scale for randomized trials: Item Response Theory and factor analyses

Emiliano Albanese, Lukas Bütikofer, Susan Armijo-Olivo, Christine Ha, Matthias Egger

## Contents

1 Item response theory (IRT) models ........ 2
- 1.1 Model specification and coefficients ........ 2
- 1.2 Factor loadings and communality ........ 6
- 1.3 Global goodness-of-fit and model comparison ........ 7
- 1.4 Item-fit and person-fit ........ 8
- 1.5 Local dependence ........ 10
- 1.6 Sensitivity analysis ........ 13

2 Reliability ........ 15

3 References ........ 17

# 1 Item response theory (IRT) models

## 1.1 Model specification and coefficients

We fitted one-parameter logistic IRT models (1PL, Supplementary table 1) and two-parameter logistic IRT models (2PL, Supplementary table 2) to the PEDro data using Stata routines `irt 1pl` and `irt 2pl`. These routines use the slope-intercept form to fit the model where the probability of study *j* with latent trait level $\theta_j$ fulfilling PEDro item *i* (i.e. answering "yes") is given by

$$P\left(Y_{ij} = 1 \,\middle|\, \alpha_i, \beta_i, \theta_j\right) = \frac{\exp\left(\alpha_i \theta_j + \beta_i\right)}{1 + \exp\left(\alpha_i \theta_j + \beta_i\right)}$$

With a common $\alpha_i = \alpha$ in case of the 1PL model. Instead of item slope $\alpha_i$ and intercept $\beta_i$ we are reporting discrimination $a_i = \alpha_i$ and difficulty $b_i = -\beta_i / \alpha_i$ from the typical IRT parametrization given by

$$P\left(Y_{ij} = 1 \,\middle|\, a_i, b_i, \theta_j\right) = \frac{\exp\left(a_i(\theta_j - b_i)\right)}{1 + \exp\left(a_i\left[\theta_j - b_i\right]\right)}$$

With a common discrimination $a_i = a$ in case of the 1PL model.

We also fitted two-dimensional two-parameter logistic IRT models (2D 2PL) using R packages *mirt*[1] (Supplementary table 3). For model comparison, we refitted 1PL and 2PL models using the same package (with results virtually identical to those obtained with Stata). *mirt* uses the slope-intercept parametrization with a scaling adjustment *D* to make the logistic metric more closely correspond to the traditional normal ogive metric. For study *j* with *m* latent trait levels $\boldsymbol{\theta}_j = (\theta_{j1}, \ldots, \theta_{jm})$ the probability fulfilling PEDro item *i* with associated slopes $\boldsymbol{\alpha}_i = (\alpha_1, \ldots, \alpha_m)$ and intercept $\beta_i$ is given by

$$P\left(Y_{ij} = 1 \,\middle|\, \boldsymbol{\alpha}_i, \beta_i, \boldsymbol{\theta}_j\right) = \frac{\exp\left(D[\boldsymbol{\alpha}_i^{\mathrm{T}} \boldsymbol{\theta}_j + \beta_i]\right)}{1 + \exp\left(D[\boldsymbol{\alpha}_i^{\mathrm{T}} \boldsymbol{\theta}_j + \beta_i]\right)}$$

In analogy to the one-dimensional models, discrimination would correspond to the slope and a latent trait-specific difficulty can be calculated by $\boldsymbol{b}_i = -\beta_i / \boldsymbol{\alpha}_i$. Difficulty for latent trait *k* corresponds to $\theta_k$ where the probability of fulfilling item *i* is 0.5 if all the other $\theta_{l \neq k}$ are 0.

Item characteristic curves, item information curves, and the test characteristic curves for the 1PL model and the 2PL model (Figures 3-5 in main manuscript and Supplementary figure 1 for a comparison of the two models) were derived using `irtgraph icc`, `irtgraph iif` and `irtgraph tcc`, respectively. Item characteristics curve for the two-dimensional 2PL model were derived using the `itemplot` function from R package *mirt* (Supplementary figure 2).

*Supplementary table 1: Difficulty and discrimination from a one-parameter logistic model of the PEDro items.*

| | Coefficient (95% CI) |
|---|---|
| **Difficulty** | |
| 1: Eligibility | -1.71 (-2.06 to -1.36) |
| 2: Random allocation | -4.01 (-4.89 to -3.13) |
| 3: Concealed allocation | 0.84 (0.57 to 1.10) |
| 4: Balanced at baseline | -1.69 (-2.03 to -1.34) |
| 5: Blinding of subjects | 3.39 (2.73 to 4.06) |
| 7: Blinding of assessors | 0.22 (-0.02 to 0.45) |
| 8: Complete follow-up | -0.71 (-0.96 to -0.45) |
| 9: Intention-to-treat | 0.85 (0.59 to 1.12) |
| 10: Between-group comparison | -3.37 (-4.04 to -2.70) |
| 11: Point and variablity measures | -2.49 (-2.96 to -2.02) |
| **Discrimination** | 1.16 (0.98 to 1.34) |

*Supplementary table 2: Difficulty and discrimination from a two-parameter logistic model of the PEDro items.*

| | Difficulty (95% CI) | Discrimination (95% CI) |
|---|---|---|
| 1: Eligibility | -1.50 (-1.98 to -1.03) | 1.43 (0.76 to 2.09) |
| 2: Random allocation | -4.02 (-7.25 to -0.80) | 1.16 (-0.05 to 2.36) |
| 3: Concealed allocation | 0.66 (0.43 to 0.89) | 1.79 (1.08 to 2.51) |
| 4: Balanced at baseline | -1.47 (-1.93 to -1.01) | 1.46 (0.78 to 2.13) |
| 5: Blinding of subjects | 3.69 (1.34 to 6.04) | 1.03 (0.20 to 1.86) |
| 7: Blinding of assessors | 0.25 (-0.02 to 0.51) | 0.99 (0.59 to 1.39) |
| 8: Complete follow-up | -1.48 (-2.53 to -0.43) | 0.45 (0.15 to 0.76) |
| 9: Intention-to-treat | 0.63 (0.42 to 0.85) | 2.05 (1.15 to 2.96) |
| 10: Between-group comparison | -9.37 (-28.16 to 9.42) | 0.36 (-0.39 to 1.11) |
| 11: Point and variablity measures | -2.94 (-4.47 to -1.40) | 0.93 (0.33 to 1.53) |

*Supplementary table 3: Coefficients from a two-dimensional two-parameter logistic IRT model of the PEDro items. The item-slope parametrization was used, as the traditional parametrization is only applicable to unidimensional models. The slopes correspond to discriminations and latent trait-specific difficulties can be derived by dividing minus intercept by slope.*

| | Item slope of first latent trait (95% CI) | Item slope of second latent trait (95% CI) | Item intercept (95% CI) |
|---|---|---|---|
| 1: Eligibility | -0.97 (-1.82 to -0.13) | -1.15 (-2.16 to -0.13) | 2.20 (1.60 to 2.79) |
| 2: Random allocation | -1.85 (-3.82 to 0.13) | 0.28 (-1.54 to 2.09) | 5.53 (2.48 to 8.57) |
| 3: Concealed allocation | -1.06 (-1.95 to -0.17) | -1.40 (-2.42 to -0.38) | -1.17 (-1.61 to -0.73) |
| 4: Balanced at baseline | -1.81 (-2.96 to -0.66) | -0.43 (-1.50 to 0.64) | 2.42 (1.54 to 3.30) |
| 5: Blinding of subjects | -9.35 (-40.12 to 21.41) | 2.84 (-9.31 to 14.99) | -18.2 (-76.7 to 40.3) |
| 7: Blinding of assessors | -1.01 (-1.59 to -0.42) | -0.39 (-1.10 to 0.32) | -0.25 (-0.51 to 0.02) |
| 8: Complete follow-up | -0.44 (-0.83 to -0.05) | -0.18 (-0.64 to 0.29) | 0.67 (0.44 to 0.91) |
| 9: Intention-to-treat | -1.46 (-3.40 to 0.47) | -2.66 (-5.32 to 0.00) | -1.69 (-2.97 to -0.41) |
| 10: Between-group comparison | -1.45 (-2.97 to 0.06) | 0.97 (-0.56 to 2.50) | 4.57 (2.30 to 6.85) |
| 11: Point and variablity measures | -1.35 (-2.27 to -0.42) | 0.00 | 3.04 (2.14 to 3.95) |

*Supplementary figure 1: Item characteristic curves for the one-parameter (1PL) and two-parameter (2PL) logistic IRT model with 95% confidence regions.*

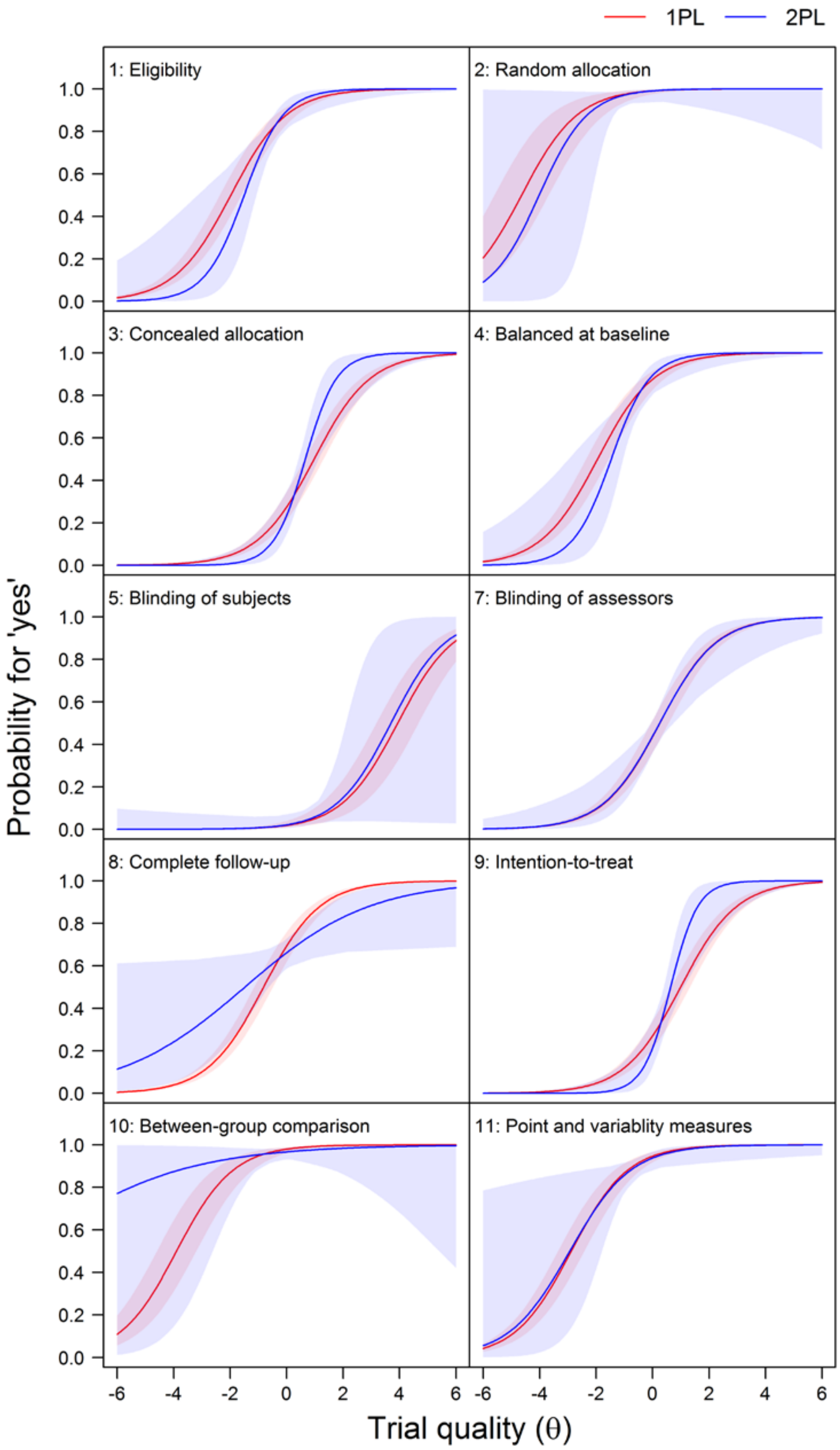

*Supplementary figure 2: Item characteristic curves for the two-dimensional two-parameter logistic model (2D 2PL). The lines represent contours at the indicated values of the probability of a positive respond to the corresponding PEDro item (0.1 to 0.9). Lines closer together represent a steep increase, i.e. a large slope. The difficulty is represented by the contour line at 0.5.*

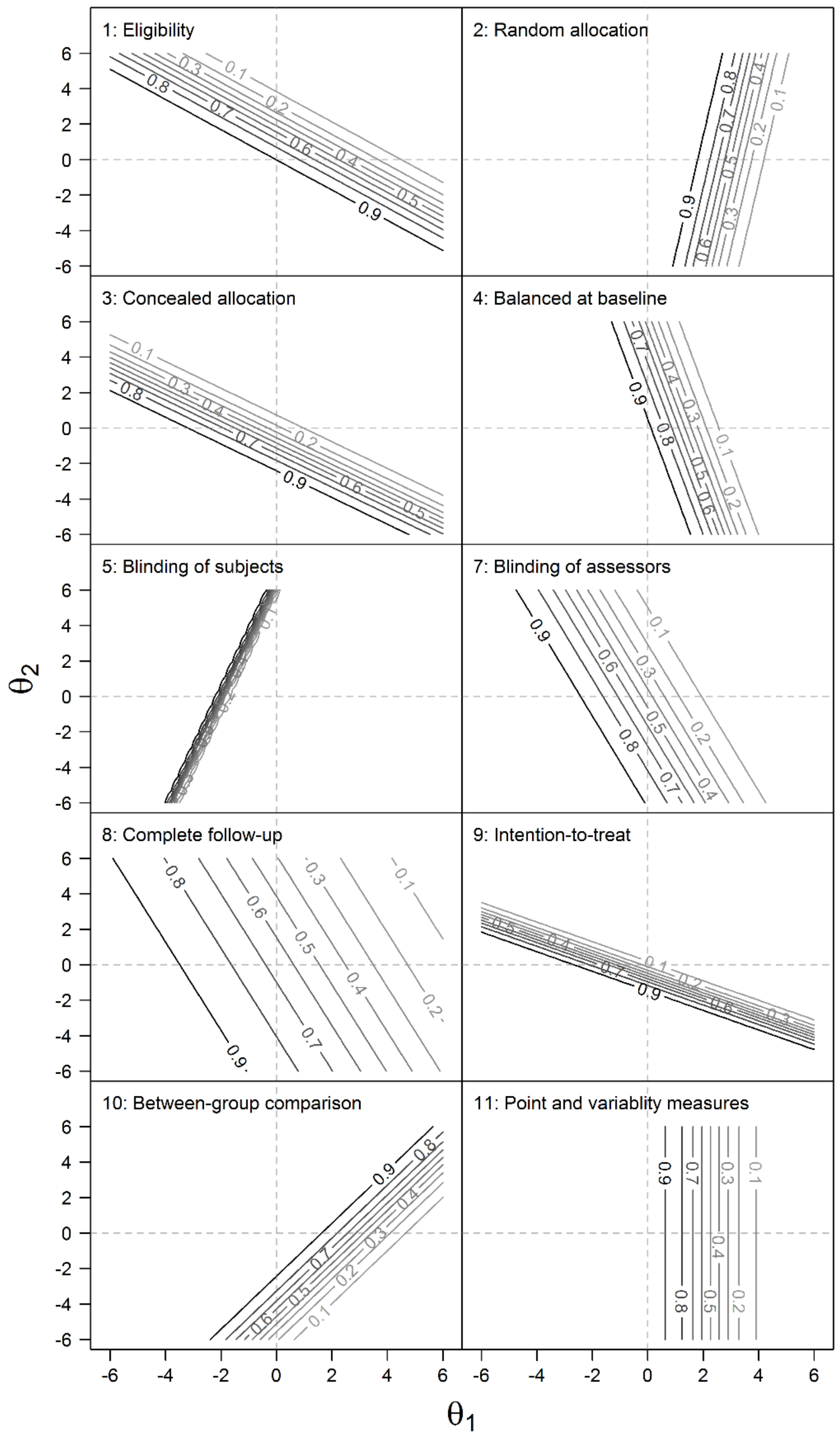

## 1.2 Factor loadings and communality

The IRT models can also be characterized by factor loadings and communality. The communality corresponds to the sum of the squared loadings and can be interpreted as the proportion of variation in an item that is explained by the factors. Items with high discrimination are reflected by large loadings and a high communality.

For the 1PL model, loadings and communality are the same for all items (as discrimination is constant) and were 0.59 and 0.34, respectively. These values are rather low, indicating that the model might not be appropriate. For the 2PL model, items 8 and 10 showed very low loadings and communalities below 0.1 (Supplementary table 4). Another four items had low communality, indicating that these items struggle to load on the single factor.

Adding a further dimension increased the loadings and the communalities (Supplementary table 5). In the two-dimensional model, items 2, 4, 5, 10 and 11 primarily loaded on the first, items 1, 3 and 9 on the second factor. Item 7 and especially 8 did not share much variance with the other items and showed cross-loading, indicating that there might be further factors.

*Supplementary table 4: Factor loadings and communality from the two-parameter logistic (2PL) model.*

| | Factor loadings | Communality |
|---|---|---|
| 1: Eligibility | 0.64 | 0.41 |
| 2: Random allocation | 0.56 | 0.32 |
| 3: Concealed allocation | 0.73 | 0.53 |
| 4: Balanced at baseline | 0.65 | 0.42 |
| 5: Blinding of subjects | 0.52 | 0.27 |
| 7: Blinding of assessors | 0.50 | 0.25 |
| 8: Complete follow-up | 0.26 | 0.07 |
| 9: Intention-to-treat | 0.77 | 0.59 |
| 10: Between-group comparison | 0.21 | 0.04 |
| 11: Point and variablity measures | 0.48 | 0.23 |

*Supplementary table 5: Factor loadings and communality from the two-dimensional two-parameter logistic (2D 2PL) model after varimax rotation. Loadings with an absolute value larger than 0.5 and 0.3 are indicated in dark and light grey, respectively.*

| | Loadings on first factor | Loadings on second factor | Communality |
|---|---|---|---|
| 1: Eligibility | -0.18 | -0.64 | 0.44 |
| 2: Random allocation | -0.71 | -0.20 | 0.55 |
| 3: Concealed allocation | -0.16 | -0.70 | 0.52 |
| 4: Balanced at baseline | -0.58 | -0.45 | 0.54 |
| 5: Blinding of subjects | -0.98 | -0.13 | 0.97 |
| 7: Blinding of assessors | -0.38 | -0.38 | 0.29 |
| 8: Complete follow-up | -0.18 | -0.19 | 0.07 |
| 9: Intention-to-treat | -0.07 | -0.87 | 0.76 |
| 10: Between-group comparison | -0.71 | 0.12 | 0.51 |
| 11: Point and variablity measures | -0.57 | -0.25 | 0.38 |

## 1.3 Global goodness-of-fit and model comparison

Global goodness-of-fit was assessed using the Akaike information criteria (AIC), the Bayesian information criteria (BIC), the M2 statistic proposed by Maydeu-Olivares[2], the root mean square error of approximation, the standardized root mean square residual, the Tucker-Lewis index (TLI, also called non-normed fit index) and the comparative fit index (CFI) (Supplementary table 6). Based on the p-value for the M2 statistic and on common thresholds of the absolute fit indices (e.g. summarized in [3], RMSEA below 0.06, SRMSR below 0.05, TLI above 0.95, CFI above 0.95) the 2PL and the 2D 2PL models showed a reasonable fit, the 1PL model did not. Comparing AIC, BIC and likelihood via likelihood ratio tests (Supplementary table 7) confirmed that the 1PL model was inferior to the 2PL and the 2D 2PL model, indicating that the assumption of a common discrimination does not hold. What is more, introducing a second dimension improved model fit considerably, suggesting that there might be more than one underlying dimension.

*Supplementary table 6: Model fit statistic of the one-parameter logistic IRT model (1PL), the two-parameter logistic IRT model (2PL) and the two-dimensional two-parameter logistic IRT model (2D 2PL).*
*AIC: Akaike information criteria, BIC: Bayesian information criteria, M2: M2 statistic[2], RMSEA: root mean square error of approximation with 95% confidence intervals (95% CI), SRMSR: standardized root mean square residual.*

| | log-likelihood | AIC | BIC | M2 (p-value) | RMSEA (95% CI) | SRMSR | Tucker-Lewis index | Comparative fit index |
|---|---|---|---|---|---|---|---|---|
| 1PL | -1364 | 2751 | 2793 | 74.2 (0.003) | 0.045 (0.021 - 0.065) | 0.066 | 0.87 | 0.87 |
| 2PL | -1351 | 2742 | 2819 | 42.8 (0.17) | 0.026 (0.000 - 0.052) | 0.048 | 0.96 | 0.97 |
| 2D 2PL | -1339 | 2735 | 2847 | 18.6 (0.85) | 0.000 (0.000 - 0.030) | 0.033 | 1.05 | 1.00 |

*Supplementary table 7: Comparison of the one-parameter logistic IRT model (1PL), the two-parameter logistic IRT model (2PL) and the two-dimensional two-parameter logistic IRT model (2D 2PL) using likelihood ratio tests.*

| | Chi2 | Degrees of freedom | P-value |
|---|---|---|---|
| 1PL vs 2PL | 26.9 | 9 | 0.001 |
| 2PL vs 2D 2PL | 24.2 | 9 | 0.004 |

### 1.4 Item-fit and person-fit

Item-fit was analyzed using function `itemfit` from R package *mirt* based on the signed chi-squared statistic[4,5] and the root mean square error of approximation (RMSEA) (Supplementary table 8). Item-fit did not much differ between the models. Items 2 and 5 with a very low proportion of negative and positive responses, respectively, showed a bad fit for both the 1PL and 2PL model, the fit for item 8 was improved in the 2PL compared to the 1PL model. The fit statistics for items 2 and 5 where not estimable for the 2D 2PL models, as the minimum cell frequencies were lower than one.

For the unidimensional models, we also calculated infit and outfit mean-square statistics[6] (Supplementary table 9 and Supplementary table 10). The former is more sensitive to observations close to an item's difficulty, the latter to observations far from an item's difficulty. The expected value of these statistics is one; values less than 1 indicate observations that are too predictable while values greater than 1 indicate unpredictability and un-modelled noise. Values between 0.5 and 1.5 are generally considered reasonable[7]. The two models were similar; item fit did not clearly improve from 1PL to 2PL model. For items 2 and 5, infit and outfit differed considerably, which is a further indication that these items are not well fitted. Items 3 and 9 show rather low values, i.e. tend to be too predictable. These two items showed almost the same parameter estimates.

We assessed person fit using function personfit from R package *mirt* based on the Zh value from Drasgow, Levine and Williams[8] (Supplementary table 11). A similar set of studies showed rather high and low values.

*Supplementary table 8: Item fit statistic for the one-parameter logistic IRT model (1PL), the two-parameter logistic IRT model (2PL) and the two-dimensional two-parameter logistic IRT model (2D 2PL). S_Chi2: signed chi-squared statistic[4,5], Df: degress of freedom, RMSEA: root mean square error of approximation. Statistics for items 2 and 5 were not estimable for the 2D 2PL model, as minimum cell frequencies of at least one could not be obtained.*

| | 1PL | | | | 2PL | | | | 2D 2PL | | | |
|---|---|---|---|---|---|---|---|---|---|---|---|---|
| | S_Chi2 | Df | P-value | RMSEA | S_Chi2 | Df | P-value | RMSEA | S_Chi2 | Df | P-value | RMSEA |
| 1: Eligibility | 3.22 | 4 | 0.52 | 0.000 | 1.73 | 3 | 0.63 | 0.000 | 2.16 | 2 | 0.34 | 0.015 |
| 2: Random allocation | 6.45 | 1 | 0.011 | 0.126 | 6.64 | 2 | 0.036 | 0.082 | not estimable | | | |
| 3: Concealed allocation | 2.37 | 4 | 0.67 | 0.000 | 2.89 | 2 | 0.24 | 0.036 | 3.13 | 3 | 0.37 | 0.011 |
| 4: Balanced at baseline | 5.01 | 4 | 0.29 | 0.027 | 3.19 | 3 | 0.36 | 0.014 | 3.39 | 2 | 0.18 | 0.045 |
| 5: Blinding of subjects | 10.73 | 2 | 0.005 | 0.113 | 10.23 | 2 | 0.006 | 0.109 | not estimable | | | |
| 7: Blinding of assessors | 6.87 | 3 | 0.08 | 0.061 | 4.64 | 3 | 0.20 | 0.040 | 4.32 | 2 | 0.12 | 0.058 |
| 8: Complete follow-up | 26.95 | 4 | <0.001 | 0.129 | 7.24 | 4 | 0.12 | 0.049 | 7.80 | 3 | 0.05 | 0.068 |
| 9: Intention-to-treat | 2.61 | 4 | 0.63 | 0.000 | 2.83 | 2 | 0.24 | 0.035 | 3.10 | 2 | 0.21 | 0.040 |
| 10: Between-group comparison | 4.71 | 3 | 0.19 | 0.041 | 5.80 | 4 | 0.21 | 0.036 | 6.59 | 3 | 0.09 | 0.059 |
| 11: Point and variablity measures | 2.95 | 4 | 0.57 | 0.000 | 3.01 | 4 | 0.56 | 0.000 | 3.78 | 3 | 0.29 | 0.028 |

*Supplementary table 9: Infit and outfit mean square statistic of the one-parameter logistic IRT model (1PL).*

| | Infit mean-square | Outfit mean-square |
|---|---|---|
| 1: Eligibility | 0.93 | 0.71 |
| 2: Random allocation | 1.23 | 0.55 |
| 3: Concealed allocation | 0.78 | 0.72 |
| 4: Balanced at baseline | 0.90 | 0.68 |
| 5: Blinding of subjects | 1.17 | 0.58 |
| 7: Blinding of assessors | 0.81 | 0.75 |
| 8: Complete follow-up | 0.95 | 0.89 |
| 9: Intention-to-treat | 0.78 | 0.70 |
| 10: Between-group comparison | 1.21 | 0.91 |
| 11: Point and variablity measures | 1.08 | 0.76 |

*Supplementary table 10: Infit and outfit mean square statistic of the two-parameter logistic IRT model (2PL).*

| | Infit mean-square | Outfit mean-square |
|---|---|---|
| 1: Eligibility | 0.90 | 0.63 |
| 2: Random allocation | 1.21 | 0.61 |
| 3: Concealed allocation | 0.72 | 0.60 |
| 4: Balanced at baseline | 0.87 | 0.66 |
| 5: Blinding of subjects | 1.12 | 0.74 |
| 7: Blinding of assessors | 0.88 | 0.83 |
| 8: Complete follow-up | 0.97 | 0.96 |
| 9: Intention-to-treat | 0.66 | 0.50 |
| 10: Between-group comparison | 1.02 | 0.97 |
| 11: Point and variablity measures | 1.04 | 0.83 |

*Supplementary table 11: Person fit for the one-parameter logistic IRT model (1PL), the two-parameter logistic IRT model (2PL) and the two-dimensional two-parameter logistic IRT model (2D 2PL) based on Zh values[8], which are on a standard normal scale. We present mean and standard deviation (sd) over all studies and the number of studies with atypical values defined as values outside the range of the 2.5% and 97.5% quantiles of the standard normal distribution (-1.96, 1.96).*

| | Zh statistic (N = 345) |
|---|---|
| 1PL | |
| mean (sd) | 0.230 (0.980) |
| atypical | 13 (3.77%) |
| 2PL | |
| mean (sd) | 0.227 (0.998) |
| atypical | 16 (4.64%) |
| 2D 2PL | |
| mean (sd) | 0.067 (1.18) |
| atypical | 20 (5.80%) |

## 1.5 Local dependence

In order to assess local independence, we used the local dependence statistic between each pair of items (a signed chi-squared value) [9] and its standardized version (Cramer's V), calculated by function `residuals` from R package *mirt* (Supplementary table 12, Supplementary table 13, Supplementary table 14)

Evidence for local dependence was found for six combinations of items in the 1PL model but only for one in the 2PL model (items 3 and 7) and for none in the 2D 2PL model.

*Supplementary table 12: A) Local dependence pairwise statistic (LD) with a p-value (P) and B) Cramer's V for the one-parameter logistic model (1PL). Potential associations with a p-value smaller than 0.05 or Cramer's V with an absolute value larger than 0.1 are indicated in grey.*

| **A)** | 1: Eligibility | | 2: Random allocation | | 3: Concealed allocation | | 4: Balanced at baseline | | 5: Blinding of subjects | | 7: Blinding of assessors | | 8: Complete follow-up | | 9: Intention-to-treat | | 10: Between-group comparison | |
|---|---|---|---|---|---|---|---|---|---|---|---|---|---|---|---|---|---|---|
| | LD | P | LD | P | LD | P | LD | P | LD | P | LD | P | LD | P | LD | P | LD | P |
| 2: Random allocation | -0.02 | 0.88 | | | | | | | | | | | | | | | | |
| 3: Concealed allocation | 1.06 | 0.30 | -0.05 | 0.83 | | | | | | | | | | | | | | |
| 4: Balanced at baseline | 0.65 | 0.42 | 0.47 | 0.49 | 1.20 | 0.27 | | | | | | | | | | | | |
| 5: Blinding of subjects | -0.09 | 0.77 | 0.07 | 0.80 | 0.07 | 0.80 | 0.85 | 0.36 | | | | | | | | | | |
| 7: Blinding of assessors | 0.76 | 0.38 | 1.74 | 0.19 | -0.07 | 0.80 | -0.09 | 0.76 | 3.16 | 0.08 | | | | | | | | |
| 8: Complete follow-up | -4.89 | 0.027 | -1.65 | 0.20 | -1.41 | 0.24 | -4.18 | 0.041 | -0.01 | 0.92 | -0.21 | 0.65 | | | | | | |
| 9: Intention-to-treat | 4.45 | 0.035 | -0.05 | 0.82 | 15.62 | <0.001 | 1.11 | 0.29 | -0.85 | 0.36 | 0.12 | 0.72 | -3.24 | 0.07 | | | | |
| 10: Between-group comparison | -0.56 | 0.45 | 0.31 | 0.58 | -7.48 | 0.006 | 0.21 | 0.65 | 0.14 | 0.71 | -2.31 | 0.13 | -0.33 | 0.56 | -3.74 | 0.05 | | |
| 11: Point and variablity measures | -2.19 | 0.14 | -0.08 | 0.78 | -0.09 | 0.76 | 2.04 | 0.15 | 0.36 | 0.55 | -0.13 | 0.72 | -4.09 | 0.043 | 0.04 | 0.84 | 0.18 | 0.67 |

| **B)** | 1: Eligibility | 2: Random allocation | 3: Concealed allocation | 4: Balanced at baseline | 5: Blinding of subjects | 7: Blinding of assessors | 8: Complete follow-up | 9: Intention-to-treat | 10: Between-group comparison |
|---|---|---|---|---|---|---|---|---|---|
| 2: Random allocation | -0.01 | | | | | | | | |
| 3: Concealed allocation | 0.06 | -0.01 | | | | | | | |
| 4: Balanced at baseline | 0.04 | 0.04 | 0.06 | | | | | | |
| 5: Blinding of subjects | -0.02 | 0.01 | 0.01 | 0.05 | | | | | |
| 7: Blinding of assessors | 0.05 | 0.07 | -0.01 | -0.02 | 0.10 | | | | |
| 8: Complete follow-up | -0.12 | -0.07 | -0.06 | -0.11 | -0.01 | -0.02 | | | |
| 9: Intention-to-treat | 0.11 | -0.01 | 0.21 | 0.06 | -0.05 | 0.02 | -0.10 | | |
| 10: Between-group comparison | -0.04 | 0.03 | -0.15 | 0.02 | 0.02 | -0.08 | -0.03 | -0.10 | |
| 11: Point and variablity measures | -0.08 | -0.01 | -0.02 | 0.08 | 0.03 | -0.02 | -0.11 | 0.01 | 0.02 |

*Supplementary table 13: A) Local dependence pairwise statistic (LD) with a p-value (P) and B) Cramer's V for the two-parameter logistic model (2PL). Potential associations with a p-value smaller than 0.05 or Cramer's V with an absolute value larger than 0.1 are indicated in grey.*

| **A)** | 1: Eligibility | | 2: Random allocation | | 3: Concealed allocation | | 4: Balanced at baseline | | 5: Blinding of subjects | | 7: Blinding of assessors | | 8: Complete follow-up | | 9: Intention-to-treat | | 10: Between-group comparison | |
|---|---|---|---|---|---|---|---|---|---|---|---|---|---|---|---|---|---|---|
| | LD | P | LD | P | LD | P | LD | P | LD | P | LD | P | LD | P | LD | P | LD | P |
| 2: Random allocation | -0.10 | 0.75 | | | | | | | | | | | | | | | | |
| 3: Concealed allocation | -0.07 | 0.79 | -0.28 | 0.60 | | | | | | | | | | | | | | |
| 4: Balanced at baseline | -0.03 | 0.85 | 0.24 | 0.63 | -0.07 | 0.79 | | | | | | | | | | | | |
| 5: Blinding of subjects | -0.11 | 0.74 | 0.07 | 0.79 | -0.00 | 0.96 | 0.79 | 0.37 | | | | | | | | | | |
| 7: Blinding of assessors | 0.59 | 0.44 | 1.96 | 0.16 | -0.73 | 0.39 | -0.20 | 0.65 | 4.10 | 0.043 | | | | | | | | |
| 8: Complete follow-up | -0.55 | 0.46 | -0.38 | 0.54 | 0.08 | 0.78 | -0.34 | 0.56 | 0.54 | 0.46 | 3.07 | 0.08 | | | | | | |
| 9: Intention-to-treat | 0.62 | 0.43 | -0.41 | 0.52 | 1.00 | 0.32 | -0.35 | 0.56 | -1.71 | 0.19 | -0.29 | 0.59 | -0.19 | 0.66 | | | | |
| 10: Between-group comparison | 0.03 | 0.85 | 1.72 | 0.19 | -2.33 | 0.13 | 2.38 | 0.12 | 0.32 | 0.57 | -0.07 | 0.79 | 0.77 | 0.38 | -0.82 | 0.37 | | |
| 11: Point and variablity measures | -1.94 | 0.16 | -0.02 | 0.90 | -0.21 | 0.65 | 2.28 | 0.13 | 0.48 | 0.49 | 0.06 | 0.81 | -0.28 | 0.60 | -0.01 | 0.92 | 2.52 | 0.11 |

| **B)** | 1: Eligibility | 2: Random allocation | 3: Concealed allocation | 4: Balanced at baseline | 5: Blinding of subjects | 7: Blinding of assessors | 8: Complete follow-up | 9: Intention-to-treat | 10: Between-group comparison |
|---|---|---|---|---|---|---|---|---|---|
| 2: Random allocation | -0.02 | | | | | | | | |
| 3: Concealed allocation | -0.01 | -0.03 | | | | | | | |
| 4: Balanced at baseline | -0.01 | 0.03 | -0.01 | | | | | | |
| 5: Blinding of subjects | -0.02 | 0.01 | -0.00 | 0.05 | | | | | |
| 7: Blinding of assessors | 0.04 | 0.08 | -0.05 | -0.02 | 0.11 | | | | |
| 8: Complete follow-up | -0.04 | -0.03 | 0.02 | -0.03 | 0.04 | 0.09 | | | |
| 9: Intention-to-treat | 0.04 | -0.03 | 0.05 | -0.03 | -0.07 | -0.03 | -0.02 | | |
| 10: Between-group comparison | 0.01 | 0.07 | -0.08 | 0.08 | 0.03 | -0.01 | 0.05 | -0.05 | |
| 11: Point and variablity measures | -0.07 | -0.01 | -0.02 | 0.08 | 0.04 | 0.01 | -0.03 | -0.01 | 0.09 |

*Supplementary table 14: A) Local dependence pairwise statistic (LD) with p-value (P)[9] and B) Cramer's V for the two-dimensional two-parameter logistic model (2D 2PL). There was no evidence for local dependencies.*

| **A)** | 1: Eligibility | | 2: Random allocation | | 3: Concealed allocation | | 4: Balanced at baseline | | 5: Blinding of subjects | | 7: Blinding of assessors | | 8: Complete follow-up | | 9: Intention-to-treat | | 10: Between-group comparison | |
|---|---|---|---|---|---|---|---|---|---|---|---|---|---|---|---|---|---|---|
| | LD | P | LD | P | LD | P | LD | P | LD | P | LD | P | LD | P | LD | P | LD | P |
| 2: Random allocation | -0.01 | 0.93 | | | | | | | | | | | | | | | | |
| 3: Concealed allocation | -0.13 | 0.72 | -0.02 | 0.89 | | | | | | | | | | | | | | |
| 4: Balanced at baseline | 0.00 | 0.95 | -0.10 | 0.75 | 0.08 | 0.78 | | | | | | | | | | | | |
| 5: Blinding of subjects | -0.13 | 0.72 | 0.02 | 0.89 | 0.20 | 0.66 | 0.07 | 0.78 | | | | | | | | | | |
| 7: Blinding of assessors | 0.77 | 0.38 | 1.33 | 0.25 | -0.13 | 0.72 | -1.39 | 0.24 | 0.96 | 0.33 | | | | | | | | |
| 8: Complete follow-up | -0.42 | 0.52 | -0.62 | 0.43 | 0.29 | 0.59 | -0.76 | 0.38 | 0.06 | 0.80 | 2.54 | 0.11 | | | | | | |
| 9: Intention-to-treat | 0.03 | 0.86 | -0.01 | 0.92 | 0.01 | 0.91 | -0.00 | 0.95 | -0.12 | 0.73 | -0.02 | 0.88 | -0.05 | 0.81 | | | | |
| 10: Between-group comparison | 0.23 | 0.63 | -0.03 | 0.86 | -0.87 | 0.35 | 0.01 | 0.91 | 0.02 | 0.89 | -1.13 | 0.29 | 0.25 | 0.62 | 0.02 | 0.88 | | |
| 11: Point and variablity measures | -1.35 | 0.24 | -0.74 | 0.39 | -0.00 | 0.97 | 0.13 | 0.71 | 0.06 | 0.81 | -0.12 | 0.72 | -0.63 | 0.43 | 0.27 | 0.61 | 0.00 | 0.96 |

| **B)** | 1: Eligibility | 2: Random allocation | 3: Concealed allocation | 4: Balanced at baseline | 5: Blinding of subjects | 7: Blinding of assessors | 8: Complete follow-up | 9: Intention-to-treat | 10: Between-group comparison |
|---|---|---|---|---|---|---|---|---|---|
| 2: Random allocation | -0.00 | | | | | | | | |
| 3: Concealed allocation | -0.02 | -0.01 | | | | | | | |
| 4: Balanced at baseline | 0.00 | -0.02 | 0.01 | | | | | | |
| 5: Blinding of subjects | -0.02 | 0.01 | 0.02 | 0.01 | | | | | |
| 7: Blinding of assessors | 0.05 | 0.06 | -0.02 | -0.06 | 0.05 | | | | |
| 8: Complete follow-up | -0.03 | -0.04 | 0.03 | -0.05 | 0.01 | 0.09 | | | |
| 9: Intention-to-treat | 0.01 | -0.01 | 0.01 | -0.00 | -0.02 | -0.01 | -0.01 | | |
| 10: Between-group comparison | 0.03 | -0.01 | -0.05 | 0.01 | 0.01 | -0.06 | 0.03 | 0.01 | |
| 11: Point and variablity measures | -0.06 | -0.05 | -0.00 | 0.02 | 0.01 | -0.02 | -0.04 | 0.03 | 0.00 |

## 1.6 Sensitivity analysis

As a sensitivity analysis, we excluded item 1 (eligibility), which is not used to calculate the overall PEDro score and fitted the same models (Supplementary table 15 - Supplementary table 19). The results were very similar to the main analysis.
For the two-dimensional model, the orientation of the item slopes is arbitrary—the inversion of the slope of the second factor compared to the main model has no meaning.

*Supplementary table 15: Coefficients from a one-parameter logistic model of the PEDro items excluding item 1.*

| | Coefficient (95% CI) |
|---|---|
| **Difficulty** | |
| 2: Random allocation | -4.06 (-4.97 to -3.15) |
| 3: Concealed allocation | 0.85 (0.58 to 1.11) |
| 4: Balanced at baseline | -1.71 (-2.06 to -1.35) |
| 5: Blinding of subjects | 3.43 (2.74 to 4.12) |
| 7: Blinding of assessors | 0.22 (-0.02 to 0.46) |
| 8: Complete follow-up | -0.71 (-0.97 to -0.46) |
| 9: Intention-to-treat | 0.86 (0.59 to 1.13) |
| 10: Between-group comparison | -3.41 (-4.10 to -2.71) |
| 11: Point and variablity measures | -2.52 (-3.01 to -2.03) |
| **Discrimination** | 1.14 (0.95 to 1.33) |

*Supplementary table16: Coefficients from a two-parameter logistic model of the PEDro items excluding item 1.*

| | Difficulty (95% CI) | Discrimination (95% CI) |
|---|---|---|
| 2: Random allocation | -3.89 (-7.01 to -0.77) | 1.21 (-0.09 to 2.51) |
| 3: Concealed allocation | 0.65 (0.42 to 0.88) | 1.87 (1.04 to 2.69) |
| 4: Balanced at baseline | -1.46 (-1.94 to -0.98) | 1.48 (0.74 to 2.21) |
| 5: Blinding of subjects | 3.53 (1.37 to 5.69) | 1.09 (0.22 to 1.97) |
| 7: Blinding of assessors | 0.26 (-0.02 to 0.54) | 0.94 (0.53 to 1.35) |
| 8: Complete follow-up | -1.42 (-2.43 to -0.42) | 0.47 (0.15 to 0.80) |
| 9: Intention-to-treat | 0.65 (0.42 to 0.88) | 1.90 (1.04 to 2.76) |
| 10: Between-group comparison | -9.32 (-28.63 to 9.99) | 0.36 (-0.41 to 1.14) |
| 11: Point and variablity measures | -2.56 (-3.72 to -1.39) | 1.12 (0.44 to 1.80) |

*Supplementary table 17: Coefficients from a two-dimensional two-parameter logistic IRT model of the PEDro items excluding item 1. The item-slope parametrization was used, as the traditional parametrization is only applicable to unidimensional models.*

| | Item slope of factor one (95% CI) | Item slope of factor two (95% CI) | Item intercept (95% CI) |
|---|---|---|---|
| 2: Random allocation | -1.78 (-3.64 to 0.09) | -0.45 (-2.35 to 1.46) | 5.47 (2.55 to 8.39) |
| 3: Concealed allocation | -1.34 (-2.49 to -0.19) | 1.60 (-0.03 to 3.23) | -1.29 (-1.97 to -0.61) |
| 4: Balanced at baseline | -1.79 (-2.88 to -0.70) | 0.23 (-0.86 to 1.32) | 2.38 (1.53 to 3.23) |
| 5: Blinding of subjects | -8.77 (-37.25 to 19.70) | -3.32 (-16.36 to 9.71) | -17.5 (-72.6 to 37.6) |
| 7: Blinding of assessors | -1.00 (-1.52 to -0.48) | 0.26 (-0.46 to 0.98) | -0.25 (-0.51 to 0.02) |
| 8: Complete follow-up | -0.46 (-0.83 to -0.09) | 0.16 (-0.36 to 0.67) | 0.68 (0.44 to 0.91) |
| 9: Intention-to-treat | -1.42 (-2.95 to 0.11) | 1.90 (-0.07 to 3.87) | -1.43 (-2.35 to -0.50) |
| 10: Between-group comparison | -1.45 (-3.19 to 0.28) | -1.37 (-3.31 to 0.58) | 4.89 (1.89 to 7.89) |
| 11: Point and variablity measures | -1.42 (-2.32 to -0.52) | 0.00 (. to .) | 3.11 (2.20 to 4.01) |

*Supplementary table 18: Model fit statistic excluding item 1. 1PL: one-parameter logistic IRT model, 2PL: the two-parameter logistic IRT model, 2D 2PL: two-dimensional two-parameter logistic IRT model.*
*AIC: Akaike information criteria, BIC: Bayesian information criteria, M2: M2 statistic[2], RMSEA: root mean square error of approximation with 95% confidence intervals (95% CI), SRMSR: standardized root mean square residual.*

| | log-likelihood | AIC | BIC | M2 (p-value) | RMSEA (95% CI) | SRMSR | Tucker-Lewis index | Comparative fit index |
|---|---|---|---|---|---|---|---|---|
| 1PL | -1223 | 2466 | 2505 | 61.0 (0.004) | 0.046 (0.021 - 0.069) | 0.065 | 0.85 | 0.86 |
| 2PL | -1213 | 2461 | 2530 | 36.3 (0.11) | 0.032 (0.000 - 0.060) | 0.049 | 0.93 | 0.95 |
| 2D 2PL | -1201 | 2454 | 2554 | 13.4 (0.82) | 0.000 (0.000 - 0.036) | 0.032 | 1.06 | 1.00 |

*Supplementary table 19: Comparison of models excluding item 1 using likelihood ratio tests. 1PL: one-parameter logistic IRT model, 2PL: two-parameter logistic IRT model, 2D 2PL: two-dimensional two-parameter logistic IRT model (2D 2PL).*

| | Chi2 | Degrees of freedom | P-value |
|---|---|---|---|
| 1PL vs 2PL | 21.0 | 8 | 0.007 |
| 2PL vs 2D 2PL | 22.8 | 8 | 0.004 |

## 2 Reliability

The item-total correlation was assessed using Pearson's correlation coefficient between the item and the total score including the item (Supplementary table 20). Some of the items showed a poor correlation to the overall PEDro score, in particular items 1, 2, 5 and 10.

The internal consistency reliability of the PEDro items was assessed using function `alpha` from R package psych[10] based on Cronbach's alpha[11] and its standardized version (based upon the correlations rather than the covariances), Guttman's lambda 6[12], the averaged between-item correlation (mean and median) and the signal-to-noise ratio[13] (Supplementary table 21). Marginal (or empirical) reliability was calculated using function `marginal_rxx` from the mirt package (Supplementary table 22).

In order to adjust for potential multidimensionality, we calculated a stratified version of Cronbach's alpha using package sirt[14]. Based on the factor loadings of the 2D 2PL model (Supplementary table 5) we assumed two (items 1, 3 and 9 vs all others) or three underlying dimensions (items 1, 3 and 9 vs 2, 5, 10 and 11 vs 4, 7 and 8). As an estimate of the total reliability of the PEDro items we also calculated McDonald's omega using function `omega` from R package psych.

The reliability between items was poor and the averaged item correlation was low.

*Supplementary table 20: Correlation between each item and the total PEDro score using Pearson's correlation coefficient with 95% confidence interval.*

| | Pearson's correlation coefficient vs total score (95% CI) |
|---|---|
| 1: Eligibility | 0.29 (0.19 - 0.38) |
| 2: Random allocation | 0.20 (0.10 - 0.30) |
| 3: Concealed allocation | 0.62 (0.55 - 0.68) |
| 4: Balanced at baseline | 0.51 (0.43 - 0.59) |
| 5: Blinding of subjects | 0.29 (0.19 - 0.38) |
| 7: Blinding of assessors | 0.60 (0.53 - 0.66) |
| 8: Complete follow-up | 0.48 (0.39 - 0.55) |
| 9: Intention-to-treat | 0.61 (0.54 - 0.68) |
| 10: Between-group comparison | 0.19 (0.09 - 0.29) |
| 11: Point and variablity measures | 0.37 (0.28 - 0.46) |

*Supplementary table 21: Reliability of the PEDro items. Cronbach's alpha below 0.6 is considered poor. The standardized alpha is based on the correlation rather than the covariance. Correlation refers to the inter-item correlation.*

| | Cronbach'a alpha (95% CI) | Standardized alpha | Guttman's lambda 6 | Mean correlation | Median correlation | Signal to noise ratio |
|---|---|---|---|---|---|---|
| All items | 0.56 (0.50 to 0.63) | 0.54 | 0.54 | 0.11 | 0.10 | 1.18 |
| If an item is dropped: | | | | | | |
| 1: Eligibility | 0.53 (0.46 to 0.60) | 0.51 | 0.50 | 0.10 | 0.10 | 1.03 |
| 2: Random allocation | 0.56 (0.50 to 0.63) | 0.54 | 0.54 | 0.12 | 0.11 | 1.19 |
| 3: Concealed allocation | 0.50 (0.42 to 0.57) | 0.49 | 0.48 | 0.10 | 0.08 | 0.95 |
| 4: Balanced at baseline | 0.52 (0.45 to 0.59) | 0.48 | 0.48 | 0.09 | 0.07 | 0.94 |
| 5: Blinding of subjects | 0.56 (0.49 to 0.62) | 0.54 | 0.53 | 0.11 | 0.11 | 1.16 |
| 7: Blinding of assessors | 0.51 (0.44 to 0.58) | 0.48 | 0.48 | 0.09 | 0.07 | 0.94 |
| 8: Complete follow-up | 0.57 (0.50 to 0.63) | 0.54 | 0.53 | 0.11 | 0.11 | 1.15 |
| 9: Intention-to-treat | 0.49 (0.42 to 0.57) | 0.48 | 0.47 | 0.09 | 0.09 | 0.94 |
| 10: Between-group comparison | 0.57 (0.50 to 0.63) | 0.55 | 0.54 | 0.12 | 0.11 | 1.23 |
| 11: Point and variablity measures | 0.55 (0.49 to 0.62) | 0.53 | 0.52 | 0.11 | 0.10 | 1.11 |

*Supplementary table 22: Marginal (or empirical) reliability from one-parameter (1PL) and two-parameter (2PL) logistic IRT models.*

| | Marginal reliability |
|---|---|
| 1PL | 0.53 |
| 2PL | 0.64 |

*Supplementary table 23: Total reliability of the PEDro items estimated by McDonald's omega assuming one, two or three underlying dimensions and stratified Cronach's alpha assuming items 1, 3 and 9 load on one dimension and the others on the second (two dimensions) or items 1, 3 and 9 load on one dimension, items 2, 5, 10 and 11 on the second and items 4, 7 and 8 on the third.*

| | McDonald's omega total | Stratified Cronbach'a alpha |
|---|---|---|
| One dimension | 0.55 | 0.56 |
| Two dimensions | 0.58 | 0.59 |
| Three dimensions | 0.62 | 0.60 |

# 3 References

[1] Chalmers RP. mirt: A multidimensional item response theory package for the R environment. Journal of Statistical Software, 2012, 48(6),1-29.

[2] Maydeu-Olivares A, Joe H. Limited information goodness-of-fit testing in multidimensional contingency tables. Psychometrika, 2006, 1;71(4), 713.

[3] Hooper D, Coughlan J, Mullen M. Structural equation modelling: Guidelines for determining model fit. Electronic Journal of Business Research Methods. 2008, 6 (1), 53-60.

[4] Orlando, M. & Thissen, D. Likelihood-based item fit indices for dichotomous item response theory models. *Applied Psychological Measurement, 24*, 2000, 50-64.

[5] Kang T, Chen TT. Performance of the generalized S-X2 item fit index for polytomous IRT models. Journal of Educational Measurement. 2008, 45(4): 391-406.

[6] Brentani, E. & Golia, S. Unidimensionality in the Rasch model: how to detect and interpret Statistica, 2007, 67, 253

[7] Linacre JM. Rasch Measurement Transactions, 2002, 16, 878

[8] Drasgow F, Levine MV, Williams EA. Appropriateness measurement with polychotomous item response models and standardized indices. British Journal of Mathematical and Statistical Psychology. 1985, 38(1), 67-86.

[9] Chen WH, Thissen D. Local dependence indexes for item pairs using item response theory. Journal of Educational and Behavioral Statistics. 1997, 22(3), 265-89.

[10] Revelle, W. psych: Procedures for Personality and Psychological Research, Northwestern University, Evanston, Illinois, USA, version 1.8.12, 2018.

[11] Cronbach, L.J. Coefficient alpha and the internal strucuture of tests. Psychometrika, 1951, 16, 297-334

[12] Guttman, L. A basis for analyzing test-retest reliability. Psychometrika, 1945, 10 (4), 255-282.

[13] Cronbach, L.J. and Gleser G.C.. The signal/noise ratio in the comparison of reliability coefficients. Educational and Psychological Measurement, 1964, 24 (3) 467-480.

[14] Robitzsch, A. sirt: Supplementary item response theory models, version 3.3-26, 2019.
